# From human-like AI to user adoption: the role of trust, attitude, and social influence in shaping behavioral intention

**DOI:** 10.3389/frai.2026.1847869

**Published:** 2026-06-17

**Authors:** Helen Helen, Mts. Arief, Amalia E. Maulana, Pantri Heriyati

**Affiliations:** Management Department, Binus Business School Doctor of Research in Management, Binus University, Jakarta, Indonesia

**Keywords:** anthropomorphic characteristic of AI, behavioral intention to use AI, customer attitude, customer trust, social influence

## Abstract

The adoption of artificial intelligence (AI) in digital banking continues to increase as financial institutions seek to improve service efficiency, personalization, and customer experience. However, user acceptance of AI-enabled banking services remains a significant challenge, particularly in high-risk financial environments where trust, security, and perceived reliability are critical considerations. Unlike conventional technology adoption studies that primarily emphasize utilitarian evaluations and social influence, this study examines how psychological and relational factors shape AI adoption in high-risk digital banking contexts. Specifically, this study investigates the influence of customer trust and anthropomorphic characteristics of AI on Behavioral Intention to Use AI-enabled digital banking services, with customer attitude acting as a mediating variable and social influence serving as a moderating variable. A quantitative approach was employed using survey data collected from 350 users of digital banking services. Data were analyzed using Partial Least Squares Structural Equation Modeling (PLS-SEM). The findings indicate that customer trust and anthropomorphic characteristics of AI positively and significantly influence both customer attitude and Behavioral Intention to Use AI-enabled banking services. Customer attitude was also found to be the strongest predictor of behavioral intention. However, the moderating effect of social influence on the relationship between customer attitude and behavioral intention was not significant. The findings suggest that AI adoption in digital banking is influenced not only by technological functionality, but also by psychological and relational factors such as trust, emotional comfort, and institutional credibility. In high-risk financial contexts, users appear to rely more heavily on personal trust evaluations and perceptions of security than on social pressure when deciding whether to adopt AI-enabled banking services. This study contributes to the AI adoption literature by demonstrating that technology adoption mechanisms in high-risk financial environments differ from conventional consumer technology contexts, where social influence is often assumed to be a dominant predictor of behavioral intention. The findings further highlight the importance of trust and human-like AI interaction in reducing uncertainty and strengthening users’ acceptance of AI-enabled banking services. Practical implications are also provided for financial institutions seeking to improve customer acceptance through transparent AI governance, trust-building strategies, and user-centered AI interaction design.

## 1 Introduction

Artificial Intelligence (AI) is rapidly transforming global industries; however, its adoption in high-risk, high-involvement sectors such as banking presents fundamentally different challenges compared to low-stakes digital service environments. Banking decisions involve substantial financial consequences, regulatory obligations, and long-term implications for users’ financial well-being (Chawande, 2025; Kumar and Tripti, 2024). Unlike AI applications in retail or entertainment contexts, AI-enabled banking systems increasingly influence sensitive activities such as credit scoring, fraud detection, loan pricing, and investment recommendations (Prema and Dhanalakshmi, 2025). Consequently, users are required to rely on automated systems under conditions characterized by uncertainty, perceived risk, algorithmic opacity, and limited transparency (Mun and Anderson, 2020). Under such conditions, AI adoption in digital banking cannot be fully explained through conventional technology adoption assumptions that primarily emphasize usefulness, efficiency, or ease of use. Instead, users may rely more heavily on psychological reassurance, institutional trust, emotional comfort, and perceptions of credibility when deciding whether to adopt AI-enabled financial technologies.

As a result, AI adoption in digital banking should be understood not merely as a technological issue, but also as a psychological and relational phenomenon shaped by trust, emotional reassurance, and perceptions of institutional credibility (Al-roshoud, 2023). AI adoption has become a strategic priority in the banking industry. Recent industry reports indicate that financial institutions are increasingly integrating generative AI technologies to improve operational efficiency, personalization, and customer experience (Kher et al., 2025). However, despite the rapid implementation of AI technologies, customer acceptance remains inconsistent, particularly in financial environments where users perceive higher levels of risk, vulnerability, and potential loss (Zhou, 2023).

Existing technology adoption literature has widely examined AI acceptance using frameworks such as the Technology Acceptance Model (TAM) and the Unified Theory of Acceptance and Use of Technology (UTAUT). However, many of these models were originally developed in relatively low-risk technology environments and implicitly assume that technology adoption mechanisms operate similarly across contexts. Such assumptions may be insufficient for explaining AI adoption in digital banking, where financial vulnerability, trust dependence, and perceived risk become substantially more salient. In high-risk financial environments, users may evaluate AI technologies not only based on functional performance, but also based on perceptions of security, institutional credibility, emotional reassurance, and confidence in automated decision-making processes.

This study argues that AI adoption in digital banking follows a fundamentally different behavioral logic from conventional consumer technology adoption. In high-risk financial environments, users are more likely to depend on institutional trust, psychological reassurance, emotional comfort, and perceived credibility than on purely utilitarian evaluations or normative social pressure. This perspective extends existing AI adoption literature by demonstrating that technology adoption mechanisms are context-dependent and may shift substantially under conditions characterized by financial risk, uncertainty, and limited transparency. Accordingly, this study challenges the assumption that technology adoption determinants operate uniformly across digital contexts and proposes that high-risk financial environments require greater consideration of psychological and relational adoption mechanisms.

In digital banking contexts, interactions are predominantly intangible and occur without direct human contact, creating substantial information asymmetry between financial institutions and customers (Nathe, 2025). Consumers often cannot directly evaluate the reliability, security, or ethical integrity of AI-driven financial systems prior to usage (Singh and Gulia, 2024). Signaling theory therefore provides an important theoretical foundation for understanding how financial institutions reduce uncertainty and communicate trustworthiness through observable signals such as cybersecurity protection, interface quality, transparency disclosures, and institutional reputation (Sharma and Klein, 2024).

These observable signals help reduce information asymmetry by enabling users to infer otherwise unobservable characteristics such as institutional competence, integrity, reliability, and technological credibility. Prior studies consistently identify trust as an important determinant of technology adoption because it reduces perceived uncertainty and vulnerability in digital environments (Kumari and Singh, 2024). However, existing studies provide inconsistent findings regarding how digital trust signals affect the adoption of AI. These inconsistent findings suggest that trust formation in AI-enabled banking cannot be fully explained through conventional technology acceptance assumptions alone, particularly in environments characterized by financial vulnerability, uncertainty, and limited algorithmic transparency (Jeyapriya and Suganthi, 2025). This inconsistency suggests that trust in AI-driven banking cannot be fully explained using conventional technology acceptance assumptions alone, particularly in contexts characterized by high perceived financial risk. Beyond institutional trust, anthropomorphic characteristics of AI may also shape user acceptance of AI-enabled banking services.

According to Media Equation Theory, individuals tend to respond socially to technologies that exhibit human-like characteristics such as empathy, conversational interaction, and personalized communication (van der Goot and Etzrodt, 2023; Sah, 2022). In high-risk financial environments, anthropomorphic AI may function as a form of psychological reassurance by reducing perceived distance between users and automated systems (Chi and Hoang Vu, 2023). Human-like AI interactions can increase perceived social presence, emotional comfort, and relational trust, thereby encouraging users to perceive AI systems as more understandable, supportive, and trustworthy (Plotkina et al., 2024; Tyulina et al., 2024). Thus, anthropomorphic AI should not be viewed merely as a design feature but as a mechanism that helps users cope with uncertainty and perceived risk in financial decision-making.

Together, signaling theory and Media Equation Theory provide a complementary framework for explaining AI adoption in high-risk digital banking environments. Signaling theory explains how financial institutions reduce uncertainty and strengthen perceptions of credibility through observable trust signals, whereas Media Equation Theory explains how human-like AI characteristics generate emotional reassurance and social responses toward automated systems. By integrating these perspectives, this study proposes that AI adoption in digital banking is shaped not only by functional technological evaluations, but also by psychological and relational mechanisms associated with trust and human-like interaction experiences.

Customer attitude also plays an important role in shaping behavioral intention toward AI-enabled banking services. Customer attitude reflects users’ overall psychological evaluation of AI-enabled banking services, including perceptions of trustworthiness, reliability, emotional comfort, convenience, and perceived risk (Santhi, 2025). Prior research consistently demonstrates that positive attitudes strengthen behavioral intention toward digital technologies. However, in AI-driven financial services, attitudes may be shaped not only by functional evaluations but also by trust perceptions and emotional responses toward AI interaction (Zhang and Fan, 2021). This suggests that customer attitude may function as an important psychological mechanism through which trust and anthropomorphic AI characteristics influence behavioral intention (Blut et al., 2021; Martin et al., 2020).

In addition to individual perceptions, prior technology adoption studies frequently emphasize the importance of social influence in shaping behavioral intention. Social influence refers to how much individuals believe that significant others support the use of a specific technology. Existing UTAUT-based studies generally assume that social endorsement strengthens technology adoption behavior. However, the role of social influence in AI-enabled banking remains theoretically uncertain. Unlike social media or entertainment technologies, financial decisions are highly personal, risk-sensitive, and associated with individual accountability. Under such conditions, users may rely more heavily on their trust evaluations and perceptions of security than on peer opinions or normative pressure. Therefore, the moderating role of social influence in high-risk AI banking contexts requires further investigation. Although prior studies have extensively examined AI adoption and digital banking, existing research continues to focus predominantly on traditional technology acceptance variables such as perceived usefulness and ease of use. Comparatively limited attention has been given to how high-risk financial environments alter the relative importance of trust, anthropomorphic AI characteristics, customer attitude, and social influence in shaping AI adoption behavior. In addition, prior studies frequently assume that social influence consistently strengthens behavioral intention across digital contexts, despite the possibility that users in financially sensitive environments may rely more heavily on personal trust evaluations than on external social pressure. Therefore, this study examines the influence of customer trust and anthropomorphic characteristics of AI on Behavioral Intention to Use AI-enabled digital banking services, with customer attitude serving as a mediating variable and social influence functioning as a moderating variable.

## 2 Literature review

### 2.1 Customer trust

Customer trust refers to users’ beliefs regarding the reliability, integrity, competence, and dependability of a service provider or technological system (Isaeva et al., 2020; Vivekananda and Meenakshi, 2024). In digital banking environments, trust becomes particularly important because financial transactions involve uncertainty, vulnerability, and perceived risk. Unlike traditional face-to-face banking interactions, AI-enabled digital banking services operate through automated systems that users cannot directly observe or fully evaluate. As a result, customers must rely on institutional signals and perceived technological credibility when deciding whether AI systems can be trusted in sensitive financial activities (Martin et al., 2020). Research suggests that trust develops progressively through repeated interactions and accumulated experiences with digital systems (Li et al., 2024). However, AI-based systems introduce additional complexity because their adaptive and autonomous characteristics may create uncertainty regarding how decisions are generated and whether those decisions are fair, transparent, and reliable (Lee, 2022; Olynick, 2024). This issue is particularly relevant in high-risk financial contexts, where algorithmic errors or system failures may produce significant financial consequences for users.

From a signaling theory perspective, trust functions as a mechanism that reduces information asymmetry between financial institutions and customers. Because users often lack the technical expertise to evaluate AI systems directly, they depend on observable trust signals such as cybersecurity protection, interface quality, transparency, institutional reputation, and responsiveness (Bach et al., 2022). These signals help customers infer whether AI-enabled banking services are secure, competent, and ethically managed. In high-risk financial environments, such trust signals become particularly important because users often cannot independently verify how AI systems generate financial decisions or manage sensitive personal data.

Although prior studies consistently identify trust as an important determinant of digital technology adoption, existing findings remain inconclusive regarding whether AI-driven financial services enhance or undermine trust perceptions. Some research indicates that AI enhances convenience, responsiveness, and service efficiency, whereas other studies suggest that excessive automation and unclear decision-making processes may foster skepticism and distrust (Jeyapriya and Suganthi, 2025). This inconsistency suggests that trust in AI-enabled banking cannot be fully explained using conventional technology acceptance assumptions alone and requires greater consideration of perceived financial risk and uncertainty.

### 2.2 Anthropomorphic characteristics of AI

Anthropomorphism refers to the attribution of human-like characteristics, emotions, and social behaviors to non-human entities such as artificial intelligence systems (Bhatti and Robert, 2023). Prior studies suggest that users tend to respond more positively to technologies that display human-like communication styles, empathy, conversational interaction, and social presence (Li and Wang, 2023). In AI-enabled service environments, anthropomorphic characteristics may reduce psychological distance between users and technology, making automated systems appear more approachable, understandable, and socially engaging (Blut et al., 2021). According to Media Equation Theory, individuals unconsciously apply social rules and interpersonal expectations when interacting with technologies that exhibit human-like attributes (Sah, 2022). This perspective is particularly relevant in digital banking contexts, where users often experience uncertainty and reduced emotional reassurance due to the absence of direct human interaction. Human-like AI interfaces may therefore function as a form of psychological reassurance that helps users cope with perceived financial risk and technological uncertainty.

Previous studies indicate that anthropomorphic AI, which refers to artificial intelligence systems designed to resemble human traits, can strengthen emotional attachment, perceived companionship, and social connectedness between users and intelligent systems (Zhang and Rau, 2022). Users might also think that anthropomorphic AI is smarter, more responsive, and better at understanding what they need (Sharma and Walia, 2023). Such perceptions are important in financial services because users are more likely to accept automated systems when they perceive them as trustworthy, socially present, and capable of providing reliable assistance during sensitive financial decision-making. Although prior research has examined anthropomorphism in AI-based services, most studies focus primarily on customer experience, enjoyment, or interaction quality in relatively low-risk service environments. Comparatively limited attention has been given to how anthropomorphic AI influences adoption behavior in high-risk financial contexts where trust, uncertainty reduction, and emotional reassurance become significantly more important.

### 2.3 Customer attitude

Customer attitude refers to users’ overall psychological evaluation of AI-enabled banking services, including perceptions of trustworthiness, reliability, emotional comfort, convenience, and perceived risk (Momani, 2020). Technology adoption literature consistently identifies attitude as a central determinant of behavioral intention because users are more likely to adopt technologies they evaluate favorably (Musawa et al., 2024). However, in AI-enabled financial services, customer attitude may be shaped not only by utilitarian evaluations but also by psychological perceptions related to trust, uncertainty, and emotional comfort (Chi and Hoang Vu, 2023). Users’ financial decisions involve personal accountability and perceived vulnerability, which heightens their sensitivity to concerns about security, transparency, and reliability (Aloumi et al., 2024). When users perceive AI systems as trustworthy, understandable, and supportive rather than merely efficient, positive attitudes toward AI-enabled banking services are likely to emerge. This perspective extends conventional technology adoption assumptions by suggesting that attitudes toward AI banking are influenced by both cognitive evaluations and emotional reassurance mechanisms (Hanji et al., 2024). Therefore, customer attitude may function as an important psychological pathway through which trust and anthropomorphic AI characteristics shape behavioral intention.

### 2.4 Social influence

Social influence refers to the extent to which individuals perceive that important others encourage or endorse the use of a particular technology (Falk et al., 2025). Traditional technology adoption models such as UTAUT propose that social influence positively affects behavioral intention because individuals tend to seek social approval, conformity, and external validation when adopting unfamiliar technologies. Prior studies suggest that social influence may reduce uncertainty in digital environments by providing shared experiences, peer recommendations, and normative reassurance (Lu et al., 2020). When users observe peers or social groups adopting AI technologies, they may perceive those technologies as more credible, trustworthy, and socially accepted. However, the role of social influence may differ substantially in high-risk financial contexts. Unlike entertainment or social media technologies, banking decisions involve personal financial responsibility, perceived risk, and privacy concerns (José et al., 2022). Under such conditions, users may rely more heavily on individual trust evaluations and perceptions of institutional reliability than on peer opinions or social endorsement. Consequently, the influence of social endorsement may weaken when users perceive that financial risks and personal consequences associated with AI adoption are too significant to delegate to collective opinion or peer recommendation. This suggests that the influence of social pressure on AI-enabled banking adoption may be weaker or more conditional than conventional technology adoption theories assume (Kang et al., 2026). Therefore, examining social influence as a moderating variable provides an opportunity to better understand whether social endorsement strengthens or weakens the relationship between customer attitude and behavioral intention in high-risk AI banking environments.

### 2.5 Behavioral intention to use

Behavioral intention refers to an individual’s willingness and planned readiness to engage in a particular behavior (Quillien and German, 2021). In technology adoption research, behavioral intention is widely regarded as a strong predictor of actual usage behavior because individuals are more likely to adopt technologies they perceive positively and consider beneficial (Conner and Norman, 2025). In the context of AI-enabled banking services, behavioral intention reflects users’ willingness to rely on automated financial technologies for activities such as transaction management, financial recommendations, fraud detection, and customer support. Prior studies indicate that users are more likely to adopt AI-enabled banking systems when they perceive them as intelligent, reliable, secure, and capable of improving financial decision-making (Adegbite, 2025; Schrank, 2025).

**Figure 1:**
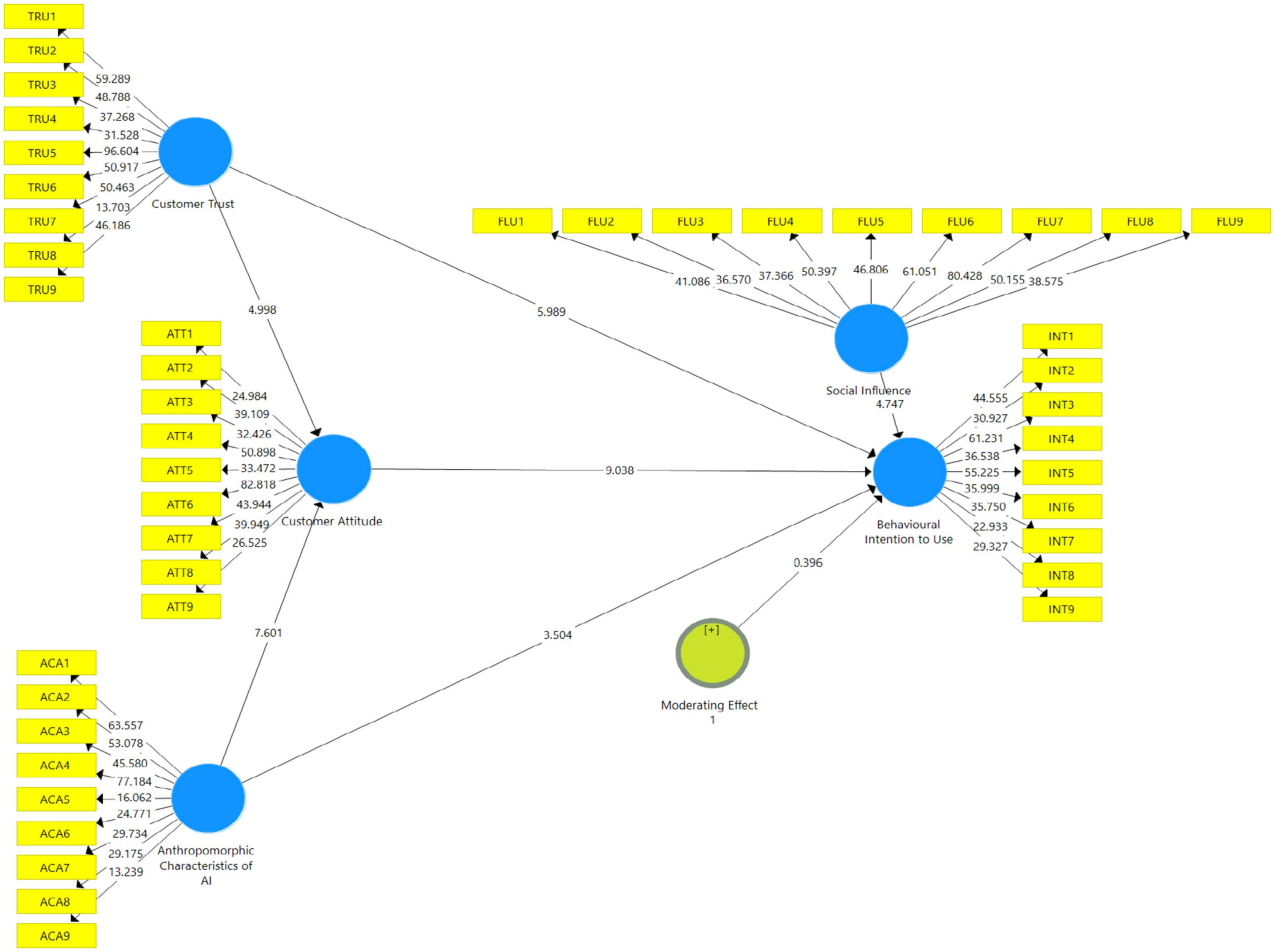
Proposed research model.

However, behavioral intention toward AI-enabled banking services may differ from intention in conventional digital technologies because financial decisions involve greater perceived risk, personal accountability, and uncertainty (Zhang and Fan, 2021). In such environments, users are more likely to evaluate AI technologies based on perceptions of trustworthiness, emotional reassurance, institutional credibility, and confidence in automated decision-making processes rather than solely on functional performance. Unlike entertainment or social media applications, AI banking systems operate in environments where errors, security failures, or algorithmic bias may directly affect users’ financial well-being (Ahmed, 2022). Consequently, behavioral intention toward AI-enabled banking is likely to depend not only on utilitarian evaluations but also on users’ perceptions of trustworthiness, emotional reassurance, and institutional credibility (Schrank, 2025). This perspective suggests that behavioral intention in high-risk AI banking contexts is shaped by a combination of technological, psychological, and relational factors rather than purely functional considerations (Atwal and Bryson, 2021) (see Figure 1).

### 2.6 Hypotheses development

Overall, the literature suggests that AI adoption in digital banking cannot be explained solely through conventional utilitarian technology adoption assumptions. In high-risk financial environments, users’ adoption decisions are likely shaped by a combination of psychological, relational, and institutional factors, including trust, emotional reassurance, social presence, and perceptions of credibility. Consequently, this study proposes an integrated framework that examines how customer trust, anthropomorphic AI characteristics, customer attitude, and social influence collectively influence behavioral intention toward AI-enabled banking services.

#### 2.6.1 Customer trust and behavioral intention to use

Trust is widely recognized as a central determinant of technology adoption because it reduces perceived uncertainty and vulnerability in digital environments (Morgan and Hunt, 1994). In financial services, trust becomes even more important because users must often rely on systems they cannot directly monitor or fully evaluate (Auerbach, 2023). AI-enabled banking services involve automated decision-making processes related to sensitive financial activities such as fraud detection, credit assessment, and financial recommendations. Under such conditions, customers are more likely to adopt AI technologies when they believe the systems are reliable, secure, transparent, and aligned with their interests (Mehndiratta et al., 2023; Temelkov, 2023). From a signaling theory perspective, trust functions as a mechanism through which users interpret institutional credibility and technological reliability. Observable signals such as security protection, transparent communication, and service responsiveness help reduce information asymmetry and strengthen confidence toward AI-enabled banking systems (Sharma and Klein, 2024). In high-risk financial environments, trust may become even more influential because users face substantial perceived consequences associated with technological failure, algorithmic bias, or misuse of sensitive financial data. Under such conditions, customers are more likely to adopt AI-enabled banking services when they perceive those systems as reliable, transparent, secure, and institutionally credible (Che et al., 2023; Lee and Chen, 2022). Therefore, trust is expected to strengthen customers’ willingness to adopt AI-enabled banking services (Noreen et al., 2023; Sami et al., 2022).

*H1*: Customer trust is positively related to Behavioral Intention to Use.

#### 2.6.2 Anthropomorphic characteristics of AI and behavioral intention to use AI

Anthropomorphism refers to the attribution of human-like characteristics and social qualities to non-human technologies (Sah, 2022). According to Media Equation Theory, individuals tend to interact socially with technologies that display human-like communication styles, emotional responsiveness, and conversational behavior (van der Goot and Etzrodt, 2023). As AI systems become more socially interactive, users may perceive them as more approachable, empathetic, and psychologically reassuring (Kim et al., 2021). In digital banking environments, anthropomorphic AI may reduce perceived uncertainty and technological anxiety by creating a sense of social presence and emotional comfort. Human-like AI interfaces can simulate interpersonal interaction, allowing users to feel more supported and understood during financial decision-making processes. This mechanism may become particularly important in digital banking environments where users seek psychological reassurance before relying on automated systems for financially sensitive decisions. Previous studies suggest that anthropomorphic AI enhances trust, emotional engagement, and perceived interaction quality, all of which contribute to stronger adoption intention (Hill and Troshani, 2024; Liu, 2025). This effect may be particularly important in high-risk financial contexts where users seek reassurance and confidence before relying on automated systems. Therefore, anthropomorphic AI characteristics are expected to positively influence behavioral intention toward AI-enabled banking services, as they can enhance user trust and comfort in using these systems.

*H2*: Anthropomorphic characteristics of AI are positively related to Behavioral Intention to Use AI.

#### 2.6.3 Customer trust and customer attitude

Customer attitude toward AI-enabled banking services is likely to be influenced by users’ trust perceptions regarding system reliability, integrity, and security (Chi and Hoang Vu, 2023; Pandey and Dangi, 2024). In digital financial environments characterized by uncertainty and limited transparency, trust functions not only as a cognitive evaluation, but also as a psychological assurance mechanism that reduces perceived risk and vulnerability (Aloumi et al., 2024; Supriandi and Supriadi, 2025). Prior studies suggest that users who perceive AI systems as trustworthy are more likely to evaluate those systems positively and develop favorable attitudes toward their use (Troshani et al., 2021). When customers believe AI-enabled banking systems operate ethically, transparently, and competently, they are more likely to perceive those technologies as beneficial and dependable. Conversely, distrust toward automated systems may generate anxiety, skepticism, and resistance toward AI adoption (Adamyk et al., 2024; Ahmed, 2022). This relationship may become stronger in high-risk financial contexts because customers often rely on trust evaluations when assessing technologies associated with financial consequences and personal accountability. Therefore, customer trust is expected to positively shape customer attitude toward AI-enabled banking services (Saxena et al., 2023; Santhi, 2025).

*H3*: Customer trust is positively related to customer attitude.

#### 2.6.4 Anthropomorphic characteristic of AI and customer attitude

Anthropomorphic AI systems incorporate human-like attributes such as conversational interaction, emotional responsiveness, and personalized communication, which means they can mimic human behaviors and emotions (Akbulut et al., 2024; Kim and Im, 2023). These characteristics may increase perceived social presence and improve users’ emotional experiences when interacting with AI technologies, leading to greater user satisfaction and engagement with these systems. Previous research indicates that anthropomorphic AI agents are often perceived as more engaging, trustworthy, and supportive, leading to more favorable customer evaluations (Przegalinska and Ciechanowski, 2019; Qiu and Benbasat, 2015). In financial service environments, where users may experience uncertainty and reduced emotional reassurance due to limited human interaction, anthropomorphic AI may help create feelings of familiarity, comfort, and interpersonal connection (Priya and Sharma, 2023). From the perspective of media equation theory, users tend to apply social expectations to technologies that display human-like behavior. Consequently, AI systems that appear socially intelligent and emotionally responsive may reduce psychological resistance toward automated banking services and strengthen positive attitudes toward their use. This mechanism is particularly relevant in high-risk banking environments where emotional reassurance and perceived trustworthiness become important determinants of customer evaluations.

*H4*: Anthropomorphic characteristics of AI are positively related to customer attitude.

#### 2.6.5 Customer attitude and behavioral intention to use

Technology adoption literature consistently identifies customer attitude as a major determinant of behavioral intention because individuals are more likely to adopt technologies they evaluate positively (Chou et al., 2020). In AI-enabled banking environments, positive customer attitudes are more likely to emerge when users perceive AI systems as trustworthy, emotionally reassuring, secure, and supportive of their financial decision-making needs. This relationship may become particularly important in high-risk financial environments where users rely heavily on psychological evaluations related to security, confidence, and emotional comfort when deciding whether to adopt AI-enabled banking services (Toros et al., 2024). Therefore, positive customer attitudes toward AI-enabled banking services are expected to strengthen Behavioral Intention to Use AI technologies in financial activities (Piotrowski and Orzeszko, 2023).

*H5*: Customer Attitude is positively related towards Behavioral Intention to Use AI.

#### 2.6.6 Moderating effect of social influence

Prior technology adoption studies generally argue that social influence strengthens behavioral intention because individuals often rely on peer opinions and social endorsement when evaluating unfamiliar technologies (Noreen et al., 2023; Yadav, 2016). Social influence may reduce uncertainty by providing external validation regarding the usefulness, credibility, and legitimacy of emerging technologies. In AI-enabled banking environments, positive social cues such as recommendations, peer experiences, and widespread adoption may reinforce users’ confidence toward AI-driven services (Kasula, 2023; Pandey and Dangi, 2024). Previous studies also suggest that social influence can strengthen technology acceptance by enhancing perceived trust and reducing psychological resistance toward automated systems (Che et al., 2023; Latikka et al., 2023; Lee et al., 2023). However, the effectiveness of social influence in financial decision-making contexts remains theoretically uncertain. Banking decisions are highly personal, risk-sensitive, and associated with individual accountability. Consequently, users may prioritize their own evaluations of trustworthiness, reliability, and security rather than relying on peer endorsement when deciding whether to adopt AI-enabled banking services. This creates an important theoretical question regarding whether social influence meaningfully strengthens the relationship between customer attitude and behavioral intention in high-risk AI banking contexts. Therefore, this study examines social influence as a moderating variable rather than assuming it functions solely as a direct predictor of behavioral intention.

*H6*: Social influence positively moderates the relationship between customer attitude and Behavioral Intention to Use AI-enabled banking services.

## 3 Methods

This study employs a quantitative explanatory research design to examine the influence of customer trust and anthropomorphic characteristics of AI on Behavioral Intention to Use AI-enabled digital banking services, with customer attitude acting as a mediating variable and social influence serving as a moderating variable. A quantitative approach was selected because it enables the objective measurement of latent constructs and facilitates the statistical testing of hypothesized relationships among variables. Primary data were collected using a structured questionnaire distributed through an online self-administered questionnaire using Google Forms between July and August 2025. The survey targeted individuals who had prior experience using digital banking services in Indonesia. Purposive sampling was employed to ensure that respondents possessed relevant experience and familiarity with AI-enabled digital banking applications. Participants included adult users of AI-enabled digital banking services, consisting of respondents within the age categories of 18–24 years, 25–35 years, 36–45 years, and above 45 years. A total of 350 valid responses were retained for analysis after data screening and completeness evaluation. The questionnaire items were adapted from previously validated scales in the literature to ensure construct validity and measurement reliability. Customer trust was measured using items adapted from (Sun and Lin, 2010; Wilson, 1990); anthropomorphic characteristics of AI and social influence were adapted from (Venkatesh et al., 2003); customer attitude was adapted from (Chawla and Joshi, 2023); and behavioral intention was adapted from (Aswathy et al., 2025). Anthropomorphic characteristics of AI were operationalized as users’ perceptions regarding the extent to which AI-enabled banking systems exhibit human-like interaction characteristics such as conversational communication, responsiveness, empathy, and personalized interaction. All measurement items were assessed using a five-point Likert scale ranging from 1 = strongly disagree to 5 = strongly agree. Prior to the main survey distribution, the questionnaire instrument underwent expert validation involving academic supervisors to evaluate content relevance, clarity, and wording appropriateness. Revisions were subsequently conducted to eliminate ambiguity, reduce overlapping meanings, and improve readability. All questionnaire items met the required validity and reliability criteria; therefore, no measurement items were removed during the analysis process. According to institutional guidelines and national regulations, formal ethical approval was not required because the study involved anonymous and voluntary survey participation without collecting sensitive personal data. Participants provided informed consent prior to participation. The study employed Partial Least Squares Structural Equation Modeling (PLS-SEM) using SmartPLS software for data analysis. PLS-SEM was selected because it is appropriate for examining complex relationships among latent variables and is widely applied in exploratory and prediction-oriented behavioral research involving technology adoption models. The selected research design was considered appropriate because the study aims to examine psychological and relational factors influencing AI adoption behavior within high-risk digital banking environments. The measurement model assessment evaluated construct reliability, convergent validity, and discriminant validity. Reliability was assessed using Cronbach’s Alpha and Composite Reliability (CR) with threshold values exceeding 0.70, while convergent validity was assessed using Average Variance Extracted (AVE) with values exceeding 0.50. Discriminant validity was evaluated using the Fornell–Larcker criterion. The structural model assessment examined the significance of the hypothesized relationships among latent variables. Bootstrapping procedures were applied to estimate path coefficients, t-statistics, and *p*-values. Although PLS-SEM does not require strict normality assumptions, data normality was preliminarily assessed through skewness and kurtosis evaluation to ensure acceptable data distribution characteristics for multivariate analysis. In addition, Variance Inflation Factor (VIF) values were evaluated to assess potential collinearity issues among constructs. Common method bias was additionally assessed using the full collinearity VIF approach, where VIF values below the recommended threshold indicated that common method bias was unlikely to pose serious concerns regarding common method bias. The structural relationships were considered significant when the t-statistic exceeded 1.96 and the p-value was below 0.05. Measurement invariance assessment was not conducted because the study did not perform subgroup or multi-group comparisons.

## 4 Results

### 4.1 Respondent profile

Table 1 presents the demographic characteristics of the respondents. The majority of respondents were above 45 years old (37%), followed by respondents aged 36–45 years (25%), indicating that the sample was largely composed of mature digital banking users with potentially greater financial decision-making experience. In terms of monthly income, most respondents reported earnings between IDR 15–19.9 million (40%), suggesting that a substantial proportion of participants belonged to middle-to-upper income groups actively engaged in digital financial services. Regarding digital banking usage, SeaBank was identified as the most frequently used platform (30%), followed by Jago (27%) and Blu (23%). These findings indicate that the respondents were familiar with contemporary digital banking platforms that increasingly integrate AI-enabled features and automated financial services.

**Table 1:** Demographic profile of respondents.

Characteristics	Category	Frequency (*N*)	Percentage (%)
Age (years old)	18–24	59	17
	25–35	74	21
	36–45	89	25
	>45	128	37
Monthly income (million IDR)	<5	21	6
	5–9.9	57	16
	10–14.9	72	21
	15–19.9	142	40
	>20	59	17
Primary digital banking platform used	Digibank	27	8
	LineBank	42	12
	Blu	81	23
	Jago	95	27
	SeaBank	105	30

### 4.2 Measurement model

The results of the measurement model test were obtained as shown in Table 2 below.

**Table 2:** Outer loading.

Indicators	ACA	Indicators	BITU	Indicators	CA	Indicators	CT	Indicators	SI
ACA1	0.859	BITU1	0.822	CA1	0.703	CT1	0.857	SI1	0.801
ACA2	0.847	BITU2	0.763	CA2	0.801	CT2	0.846	SI2	0.762
ACA3	0.845	BITU3	0.836	CA3	0.761	CT3	0.810	SI3	0.805
ACA4	0.880	BITU4	0.792	CA4	0.849	CT4	0.780	SI4	0.822
ACA5	0.655	BITU5	0.838	CA5	0.776	CT5	0.899	SI5	0.828
ACA6	0.736	BITU6	0.777	CA6	0.885	CT6	0.850	SI6	0.854
ACA7	0.788	BITU7	0.792	CA7	0.822	CT7	0.852	SI7	0.886
ACA8	0.789	BITU8	0.730	CA8	0.807	CT8	0.600	SI8	0.846
ACA9	0.593	BITU9	0.756	CA9	0.748	CT9	0.822	SI9	0.794

Anthropomorphic characteristic of AI (ACA); social influence (SI); behavioral intention to use (BITU), customer trust (CT), customer attitude (CA).

Table 2 shows the outer loading values for all indicators used in this study. Most indicators have loading values above 0.70, indicating that the items are reliable and able to measure their respective constructs appropriately. According to (Hair et al., 2017), outer loading values above 0.70 indicate good indicator reliability. Several indicators, such as ACA5 (0.655), ACA9 (0.593), and CT8 (0.600), showed values slightly below 0.70. However, these indicators were retained because the overall Composite Reliability (CR) and Average Variance Extracted (AVE) values for each construct remained above the recommended thresholds. Therefore, all constructs still demonstrated adequate convergent validity and reliability. Overall, the results indicate that all measurement items adequately represent their respective constructs, and no indicators were removed from the analysis.

Table 3 presents the results of construct reliability and convergent validity assessment. The Cronbach’s Alpha (*α*) values for all constructs range from 0.921 to 0.940, exceeding the recommended threshold of 0.70, which indicates good internal consistency reliability. Similarly, the Composite Reliability (CR) values range from 0.933 to 0.950, confirming that all constructs have satisfactory reliability. In addition, the Average Variance Extracted (AVE) values for all constructs range from 0.612 to 0.677, which are above the recommended threshold of 0.50. This indicates that each construct explains more than 50% of the variance of its indicators, demonstrating adequate convergent validity. Overall, the results confirm that all constructs used in this study are reliable and valid for further analysis.

**Table 3:** Construct reliability.

Variables	Indicator		
	*α*	CR	AVE
ACA	0.921	0.933	0.612
BITU	0.924	0.937	0.624
CA	0.927	0.939	0.634
CT	0.938	0.947	0.668
SI	0.940	0.950	0.677

Anthropomorphic characteristic of AI (ACA); social influence (SI); behavioral intention to use (BITU), customer trust (CT), customer attitude (CA).

Cronbach’s alpha (*α*); composite reliability (CR); average variance extracted (AVE).

Table 4 presents the results of discriminant validity assessment using the HTMT ratio and Fornell–Larcker criterion. The HTMT values for all construct pairs are below the recommended threshold of 0.90, indicating that each construct is empirically distinct from the others. In addition, based on the Fornell–Larcker criterion, the square root of the AVE values for each construct is greater than the correlation values with other constructs. This indicates that each construct shares more variance with its own indicators than with other constructs in the model. Overall, the results confirm that the measurement model demonstrates adequate discriminant validity.

**Table 4:** Discriminant validity assessment.

HTMT						Fornell–Larcker criterion			
Variables	ACA	BITU	CA	CT	SI	BITU	CA	CT	SI
ACA									
BITU	0.398					*0.79*			
CA	0.378	0.646				0.602	*0.796*		
CT	0.151	0.419	0.28			0.409	0.278	*0.817*	
SI	0.217	0.454	0.402	0.147		0.433	0.382	0.143	*0.823*

The italicized diagonal values represent the square root of the Average Variance Extracted (√AVE) for each construct.

It can be seen that All VIF values were below the recommended threshold of 5, indicating that multicollinearity was not a concern. Furthermore, full collinearity VIF values below 3.3 suggest that common method bias was unlikely to threaten the validity of the model (see Table 5).

**Table 5:** Collinearity statistics (VIF).

Variables	BITU	CA
ACA	1.183	1.021
BITU		
CA	1.377	
CT	1.087	1.021
SI	1.184	

Anthropomorphic characteristic of AI (ACA); social influence (SI); behavioral intention to use (BITU), customer trust (CT), customer attitude (CA).

Table 6 presents the R Square (*R*^2^) and Adjusted R Square values for the endogenous variables in the structural model. The *R*^2^ value for Behavioral Intention to Use is 0.491, indicating that customer trust, anthropomorphic characteristics of AI, customer attitude, and social influence explain 49.1% of the variance in Behavioral Intention to Use AI-enabled banking services. Based on Hair et al. (2017), this value can be interpreted as moderate explanatory power. Meanwhile, the *R*^2^ value for Customer Attitude is 0.196, indicating that customer trust and anthropomorphic characteristics of AI explain 19.6% of the variance in customer attitude. This value suggests a weak to moderate level of explanatory power. Overall, the results indicate that the proposed model has acceptable predictive capability in explaining users’ attitudes and behavioral intention toward AI-enabled digital banking services.

**Table 6:** Coefficient of determination (*R*^2^).

Variables	*R* square	*R* square adjusted
BITU	0.491	0.484
CA	0.196	0.191

Table 7 presents the effect size (*f*^2^) values of the exogenous variables on the endogenous constructs. According to Hair et al. (2017), *f*^2^ values of 0.02, 0.15, and 0.35 indicate small, medium, and large effect sizes, respectively. The results show that Customer Attitude (CA) has a medium effect on Behavioral Intention to Use (BITU) with an *f*^2^ value of 0.219, indicating that customer attitude is the strongest predictor of behavioral intention in the model. Customer Trust (CT) has a small effect on Behavioral Intention to Use (*f*^2^ = 0.108) and a small effect on Customer Attitude (*f*^2^ = 0.063). Similarly, Anthropomorphic Characteristics of AI (ACA) have a small effect on Behavioral Intention to Use (*f*^2^ = 0.042) and a medium effect on Customer Attitude (*f*^2^ = 0.147), suggesting that anthropomorphic AI contributes more strongly to shaping customer attitudes than direct behavioral intention. Social Influence (SI) shows a small effect on Behavioral Intention to Use (*f*^2^ = 0.074), indicating a relatively limited contribution to the model. Overall, the findings suggest that customer attitude plays the most substantial role in influencing behavioral intention toward AI-enabled banking services.

**Table 7:** Effect size (*f*^2^).

Variables	BITU	CA
ACA	0.042	0.147
BITU		
CA	0.219	
CT	0.108	0.063
SI	0.074	

Table 8 presents the predictive relevance (*Q*^2^) values of the endogenous constructs. The *Q*^2^ values were calculated using the blindfolding procedure to evaluate the predictive capability of the structural model. According to (Hair et al., 2017), *Q*^2^ values greater than zero indicate that the model has predictive relevance. The results show that Behavioral Intention to Use has a *Q*^2^ value of 0.241, while Customer Attitude has a *Q*^2^ value of 0.091. These findings indicate that the structural model demonstrates adequate predictive relevance for explaining customer attitude and behavioral intention toward AI-enabled digital banking services.

**Table 8:** Predictive relevance (*Q*^2^).

Variables	*R* ^2^	*Q* ^2^
BITU	0.491	0.241
CA	0.196	0.091

Table 9 presents the model fit assessment results. According to (Hair et al., 2017), an SRMR value below 0.08 indicates acceptable model fit in PLS-SEM analysis. The results show that the SRMR value of the saturated model is 0.061, which is below the recommended threshold, indicating that the proposed model demonstrates acceptable fit with the observed data.

**Table 9:** Model fit assessment.

Model fit indices	Saturated model	Estimated model
SRMR	0.061	0.081
d_ULS	3.904	6.711
d_G	0.997	1.024
Chi-square	2115.938	2133.938
NFI	0.831	0.829

Table 10 presents the results of hypothesis testing. The findings show that Customer Trust has a positive and significant effect on Behavioral Intention to Use (*β* = 0.335, *p* < 0.001), supporting H1. Anthropomorphic Characteristics of AI also positively influence Behavioral Intention to Use (*β* = 0.295, *p* < 0.001), supporting H2. Customer Trust significantly affects Customer Attitude (*β* = 0.227, *p* < 0.001), supporting H3. Similarly, Anthropomorphic Characteristics of AI positively influence Customer Attitude (*β* = 0.348, *p* < 0.001), supporting H4. In addition, Customer Attitude has a positive and significant effect on Behavioral Intention to Use (*β* = 0.392, *p* < 0.001), supporting H5. Among all relationships, Customer Attitude demonstrates the strongest influence on Behavioral Intention to Use. However, the moderating effect of Social Influence on the relationship between Customer Attitude and Behavioral Intention to Use is not significant (*β* = −0.014, *p* = 0.713). Therefore, H6 is not supported.

**Table 10:** Hypothesis testing results.

Hypothesis	Constructs correlation	Path coefficient	T statistics	*p*-values
H1	CT→BITU	0.335	8.267	0.000
H2	ACA→BITU	0.295	7.386	0.000
H3	CT→CA	0.227	5.008	0.000
H4	ACA→CA	0.348	8.257	0.000
H5	CA→BITU	0.392	8.435	0.000
H6	CA*SI→BITU	−0.014	0.368	0.713

Anthropomorphic characteristic of AI (ACA); social influence (SI); behavioral intention to use (BITU), customer trust (CT), customer attitude (CA).

**Figure 2:**
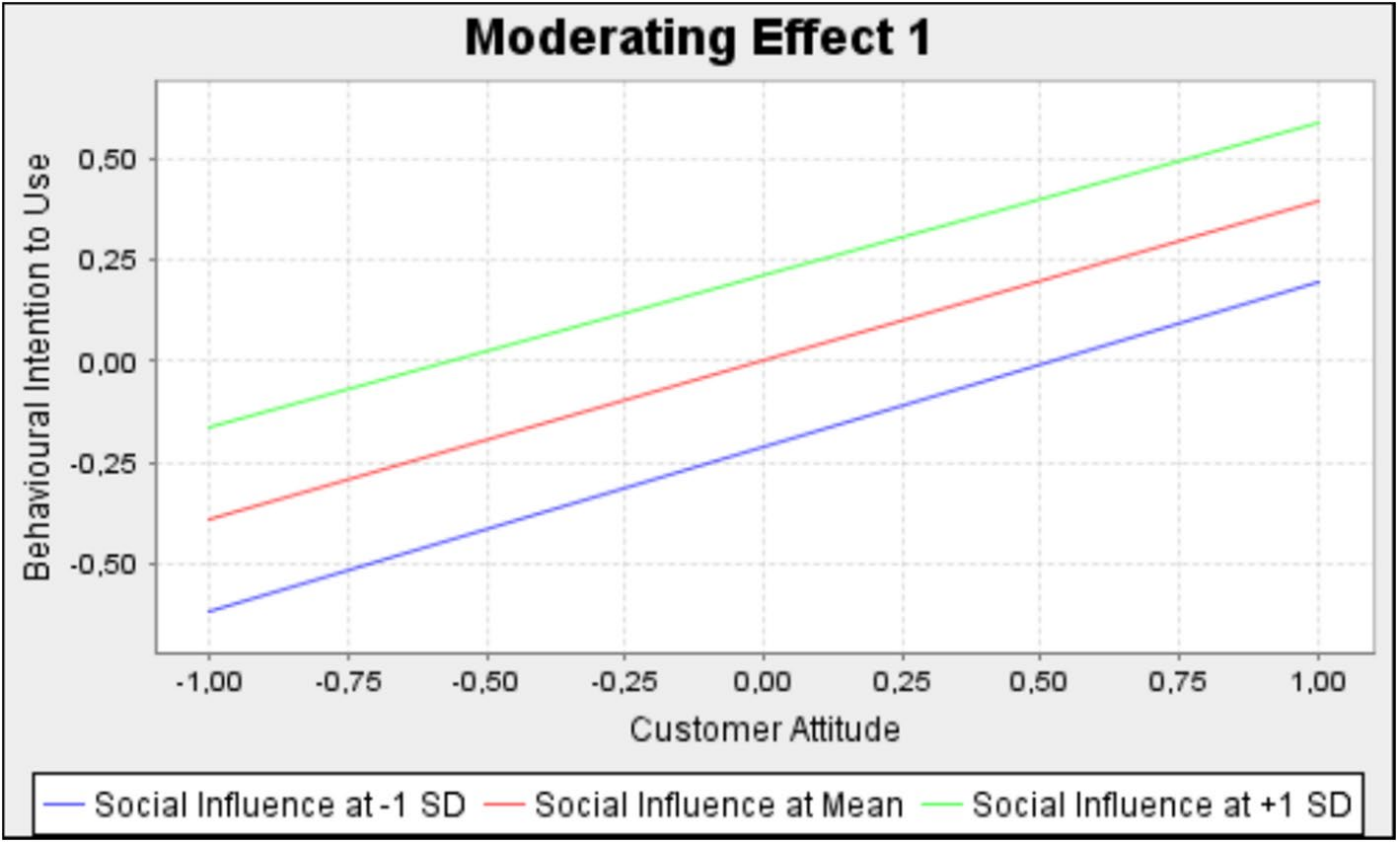
Moderating effect.

Figure 2 illustrates the moderating effect of Social Influence on the relationship between Customer Attitude and Behavioral Intention to Use AI-enabled banking services. The interaction plot shows relatively parallel slopes across low, medium, and high levels of social influence, indicating that social influence does not substantially strengthen or weaken the relationship between customer attitude and behavioral intention. This result is consistent with the hypothesis testing results, where the moderating effect of social influence was found to be insignificant (*β* = −0.014, *t* = 0.368, *p* = 0.713).

## 5 Discussion

The findings of this study suggest that AI adoption in digital banking is shaped not only by technological functionality, but also by psychological evaluations and institutional trust in high-risk financial environments. Unlike conventional technology adoption contexts where utilitarian evaluations and social influence are often dominant, AI-enabled banking services involve financial uncertainty, perceived risk, and limited transparency regarding algorithmic decision-making. Under such conditions, users appear to rely more heavily on trust evaluations, emotional comfort, and perceptions of technological credibility when forming behavioral intentions toward AI-enabled banking services.

### 5.1 Customer trust and behavioral intention to use

The findings show that customer trust has a positive and significant effect on Behavioral Intention to Use AI-enabled digital banking services, supporting H1 (*β* = 0.335, *p* < 0.001). This finding suggests that trust plays a central role in encouraging users to adopt AI-enabled banking technologies, particularly in high-risk financial environments where automated systems manage sensitive financial information and decision-making processes. In digital banking contexts, users often cannot directly evaluate how AI systems process financial data, assess risk, or generate automated recommendations. As a result, customers rely heavily on perceptions of security, reliability, and institutional credibility when deciding whether to adopt AI-enabled banking services.

The findings further indicate that trust functions as an important mechanism for reducing uncertainty, psychological resistance, and perceived complexity associated with AI-driven financial services (Adegbite, 2025; Lee and Chen, 2022). AI-enabled banking systems often involve predictive analytics, automated recommendations, and algorithmic decision-making processes that customers may perceive as difficult to understand or evaluate (Başal, 2025). Under such conditions, users are more likely to adopt AI technologies when they perceive the systems as trustworthy, secure, transparent, and ethically managed. These findings indicate that trust functions not only as a technological evaluation mechanism, but also as a psychological foundation that enables users to engage with AI-enabled banking services despite uncertainty and limited algorithmic transparency.

### 5.2 Anthropomorphic characteristics of AI and behavioral intention to use

The findings indicate that anthropomorphic characteristics of AI have a positive and significant effect on Behavioral Intention to Use AI-enabled digital banking services, supporting H2 (*β* = 0.295, *p* < 0.001). This finding suggests that AI systems with human-like characteristics, such as conversational interaction, personalized responses, and empathetic communication styles, can encourage users to adopt AI-enabled banking technologies. In digital banking environments where customers interact primarily through automated systems, human-like AI features may help reduce the perceived distance between users and technology. The results further suggest that anthropomorphic AI characteristics contribute to users’ emotional comfort and engagement when interacting with AI-enabled banking services. In high-risk financial environments, customers may feel uncertain or hesitant about relying on automated systems to manage sensitive financial activities. Human-like communication patterns and socially interactive AI features may therefore increase users’ confidence.

This finding supports Media Equation Theory, which suggests that individuals tend to respond socially and emotionally to technologies that exhibit human-like behaviors and communication styles. Another possible explanation is that anthropomorphic AI enhances users’ perceptions of social presence during digital interactions. AI systems that simulate natural communication through contextual understanding, responsiveness, and conversational dialogue may create interactions that feel more personal and socially meaningful (Liu et al., 2025). As a result, users may perceive AI-enabled banking systems as more supportive, intelligent, and capable of understanding their needs, which positively influences behavioral intention toward AI adoption. This finding is consistent with previous studies suggesting that anthropomorphic AI can improve user engagement, perceived friendliness, and interaction quality (Cuadra et al., 2024; Hanji et al., 2024).

The findings also suggest that anthropomorphic AI characteristics may reduce perceived complexity and technological anxiety associated with AI-driven financial technologies. Automated financial systems often involve algorithmic processes that users may perceive as difficult to understand (Nain and Rajan, 2024). Human-like interaction cues may simplify communication and make AI systems appear more intuitive and emotionally accessible, thereby encouraging users to engage more comfortably with AI-enabled banking services (Huang and Liang, 2025). These findings indicate that anthropomorphic AI characteristics are not merely interface-related design elements, but important relational mechanisms that strengthen users’ acceptance of AI technologies in high-involvement financial contexts. In digital banking environments characterized by uncertainty and limited human interaction, anthropomorphic AI may help financial institutions create more engaging, trustworthy, and psychologically comfortable service experiences for customers.

### 5.3 Customer trust and customer attitude

The findings indicate that customer trust has a positive and significant effect on customer attitude toward AI-enabled digital banking services, supporting H3 (*β* = 0.227, *p* < 0.001). This finding suggests that trust not only directly influences users’ behavioral intention, but also shapes how customers psychologically evaluate AI-enabled banking technologies. When users perceive AI systems as reliable, secure, and trustworthy, they are more likely to develop favourable attitudes toward the use of AI in financial services. In digital banking environments, customers often face limited transparency regarding how AI systems process financial data and generate automated decisions. As a result, trust becomes an important evaluative mechanism that helps users feel more confident and comfortable when interacting with AI-driven financial technologies. This finding is consistent with previous studies suggesting that trust positively influences users’ perceptions and evaluations of technology-based services (Li et al., 2021; Sullivan and Weger, 2025).

The findings also suggest that trust reduces psychological resistance toward AI-enabled financial systems. AI technologies in banking frequently involve automated processes such as fraud detection, credit scoring, and personalized financial recommendations, which users may perceive as complex or difficult to understand (Hossain et al., 2025). When customers trust the institution and the technology, they may become more willing to accept algorithmic decision-making and evaluate AI-enabled services more positively. This finding reinforces the role of trust as a mechanism that reduces uncertainty and strengthens users’ acceptance of automated financial technologies. Another possible explanation is that trust strengthens perceptions of institutional credibility and technological legitimacy. Financial institutions that demonstrate transparency, ethical AI governance, and reliable data protection practices are more likely to convince customers that AI systems operate in their best interests (Amin et al., 2025). Under such conditions, users may perceive AI-enabled banking services as safer, more beneficial, and more trustworthy, which positively shapes customer attitudes toward the technology. These findings indicate that customer trust is not only important for encouraging behavioral intention, but also plays a central role in shaping users’ overall evaluations and acceptance of AI-enabled banking services in high-risk financial environments.

### 5.4 Anthropomorphic characteristics of AI and customer attitude

The findings show that anthropomorphic characteristics of AI have a positive and significant effect on customer attitude toward AI-enabled digital banking services, supporting H4 (*β* = 0.348, *p* < 0.001). This finding suggests that AI systems with human-like interaction features can positively shape users’ perceptions and emotional evaluations of AI-enabled banking technologies. Features such as conversational communication, personalized responses, empathy, and natural interaction styles may make AI systems appear more socially engaging, approachable, and easier to use. The results further indicate that anthropomorphic AI characteristics contribute to users’ emotional comfort when interacting with automated financial systems. In digital banking environments where direct human interaction is limited, human-like AI features may help reduce feelings of uncertainty and impersonality during financial transactions. This finding supports previous studies suggesting that anthropomorphic AI can improve user engagement, interaction quality, and perceived friendliness in digital service environments (Mohammed, 2024; Mordi et al., 2025). Another possible explanation is that anthropomorphic AI increases users’ perceptions of social presence. AI systems that simulate human communication through contextual understanding, conversational dialogue, and responsive interactions may create experiences that feel more natural and socially meaningful (Liu et al., 2025). As a result, users may perceive AI-enabled banking services as more supportive, understandable, and trustworthy, which positively influences their attitudes toward the technology. Unlike the direct effect on behavioral intention, this finding suggests that anthropomorphic AI primarily shapes users’ internal psychological evaluations before influencing actual adoption intention.

The findings also suggest that anthropomorphic AI may help reduce perceived technological complexity and anxiety associated with AI-driven financial services. Automated financial technologies often involve algorithmic processes that users may find difficult to understand or evaluate (Sun and Reani, 2024). Human-like interaction cues may simplify communication and make AI systems appear more intuitive and emotionally accessible, thereby encouraging more positive evaluations toward AI-enabled banking services (Lee and Kim, 2025). These findings reinforce the relevance of Media Equation Theory, which explains that users tend to respond socially and emotionally to technologies that exhibit human-like characteristics. In high-risk financial contexts, anthropomorphic AI characteristics appear to function as important relational mechanisms that strengthen users’ acceptance and positive evaluations of AI-enabled banking technologies.

### 5.5 Customer attitude and behavioral intention to use

The findings indicate that customer attitude has a positive and significant effect on Behavioral Intention to Use AI-enabled digital banking services, supporting H5 (*β* = 0.392, p < 0.001). Among all the relationships examined in this study, customer attitude demonstrates the strongest influence on behavioral intention, indicating that users’ overall evaluations toward AI-enabled banking services play a central role in shaping adoption intentions. This finding suggests that users are more likely to adopt AI-enabled banking technologies when they perceive the systems as trustworthy, emotionally reassuring, secure, and supportive of their financial activities. The results further indicate that AI adoption in financial services is not determined solely by technological functionality, but also by users’ broader psychological evaluations regarding trustworthiness, emotional comfort, and perceived security. This finding is consistent with previous studies emphasizing the important role of customer attitude in shaping behavioral intention toward technology adoption (Chou et al., 2020; Musawa et al., 2024).

Another possible explanation is that customer attitude functions as an important psychological pathway linking users’ perceptions of AI technologies to behavioral intention. In this study, customer trust and anthropomorphic characteristics of AI significantly contribute to the formation of positive customer attitudes, which subsequently strengthen users’ intention to adopt AI-enabled banking services. This suggests that behavioral intention is formed not only through objective evaluations of technological capabilities, but also through subjective emotional and cognitive assessments developed during users’ interactions with AI systems. The strong role of customer attitude may also reflect the high-involvement nature of financial decision-making, where users place greater emphasis on personal evaluations related to trust, security, emotional comfort, and financial consequences when deciding whether to rely on AI-enabled banking (Ali et al., 2022; Samad et al., 2021).

### 5.6 Moderating effect of social influence

Contrary to expectations, social influence does not significantly moderate the relationship between customer attitude and Behavioral Intention to Use AI-enabled digital banking services, therefore H6 is not supported (*β* = −0.014, *p* = 0.713). This finding suggests that behavioral intention toward AI-enabled banking services may be shaped more strongly by users’ individual evaluations and trust perceptions than by external social pressure or peer influence. The insignificant moderating effect indicates that customers tend to rely on their own judgments regarding security, trustworthiness, institutional credibility, and perceived reliability when making adoption decisions toward AI-enabled banking services. Unlike social media, entertainment platforms, or lifestyle applications where peer influence often shapes behavioral intention, digital banking involves sensitive financial information, transaction security, and personal financial consequences (Vousinas and Saluja, 2025). As a result, customers may prefer to independently evaluate AI-enabled banking services before deciding to adopt them.

This finding also suggests that the role of social influence in AI adoption may vary depending on contextual risk characteristics. Traditional technology adoption models such as UTAUT generally assume that social influence consistently strengthens behavioral intention across digital environments. However, the results of this study indicate that in high-involvement financial contexts, behavioral intention may depend more heavily on internal psychological evaluations such as trust, perceived security, and emotional comfort than on external social endorsement.

This finding extends existing technology adoption literature by demonstrating that AI adoption mechanisms do not operate uniformly across digital contexts. In high-risk financial environments, behavioral intention appears to depend more heavily on internal psychological evaluations such as trust, perceived security, emotional comfort, and institutional credibility than on external social endorsement or peer influence. Another possible explanation is the increasing maturity of digital banking ecosystems. As AI-enabled banking services become more familiar and integrated into everyday financial activities, customers may develop independent perceptions and experiences regarding the technology (Akbulut et al., 2024). Under such conditions, users may rely less on peer recommendations or social approval because their behavioral intentions are shaped more strongly by direct interaction experiences and personal evaluations of AI-enabled banking services (Hildebrand and Bergner, 2021). The interaction plot analysis also supports the insignificant moderating effect, showing that different levels of social influence do not substantially change the relationship between customer attitude and behavioral intention. This finding reinforces the argument that AI adoption in digital banking follows a different behavioral logic from conventional consumer technology adoption, where social influence is often a dominant predictor of behavioral intention. Overall, these findings suggest that social influence may have a more limited role in high-risk AI-enabled financial environments, where users prioritize trust, credibility, and personal evaluations over external social pressure when deciding whether to adopt AI-enabled banking technologies.

## 6 Conclusion

This study examined the determinants of Behavioral Intention to Use artificial intelligence (AI) in digital banking by analyzing the roles of customer trust, anthropomorphic characteristics of AI, customer attitude, and social influence. Using a quantitative approach and PLS-SEM analysis on data collected from 350 digital banking users, the study provides empirical evidence regarding the factors that shape users’ acceptance of AI-enabled banking services. The findings demonstrate that customer trust and anthropomorphic characteristics of AI significantly influence Behavioral Intention to Use AI-enabled digital banking services. Both variables also positively affect customer attitude, which was found to be the strongest predictor of behavioral intention. These findings indicate that users’ adoption of AI-enabled banking technologies is shaped not only by technological functionality, but also by psychological evaluations related to trust, emotional comfort, perceived security, and interaction experience. In digital banking environments characterized by uncertainty, information asymmetry, and financial risk, trust becomes an essential mechanism that reduces users’ concerns regarding AI-driven financial decision-making. Similarly, anthropomorphic AI characteristics such as conversational interaction, personalized responses, and human-like communication styles help create more engaging and emotionally comfortable interactions between users and AI-enabled banking systems.

Another important finding is that social influence does not significantly moderate the relationship between customer attitude and Behavioral Intention to Use AI-enabled banking services. This suggests that AI adoption in high-risk financial environments may follow a different behavioral logic from conventional technology adoption contexts, where social influence is often assumed to play a dominant role. In AI-enabled banking services, users appear to rely more heavily on their own evaluations regarding trustworthiness, security, reliability, and institutional credibility rather than on peer influence or social pressure when making adoption decisions. The study therefore contributes to AI adoption literature by demonstrating that technology adoption mechanisms do not operate uniformly across digital contexts. In high-risk financial environments such as digital banking, users rely more heavily on psychological and relational evaluations, including trust, emotional comfort, and institutional credibility, than on conventional utilitarian adoption assumptions emphasized in models such as TAM and UTAUT. The findings further support the relevance of signaling theory and Media Equation Theory in explaining how institutional trust signals and human-like AI characteristics help reduce uncertainty and strengthen users’ acceptance of AI-enabled financial technologies.

From a practical perspective, the findings suggest that financial institutions should prioritize trust-building mechanisms, transparent AI governance, data security, and ethical AI implementation to strengthen customer confidence in AI-enabled banking services. In addition, banks and fintech companies should design AI systems with human-like interaction capabilities that enhance users’ emotional comfort and interaction quality. The findings also indicate that strategies emphasizing security, credibility, personalization, and positive user experience may be more effective than relying primarily on social endorsement or peer influence in encouraging AI adoption within digital banking environments.

### 6.1 Theoretical implications

This study contributes to the growing literature on artificial intelligence (AI) adoption in digital banking by extending existing technology adoption research through the integration of customer trust, anthropomorphic characteristics of AI, customer attitude, and social influence. Unlike conventional technology adoption models such as TAM and UTAUT that mainly emphasize utilitarian evaluations and social influence, this study demonstrates that AI adoption in high-risk financial environments is strongly influenced by psychological and relational factors, particularly trust, emotional comfort, and institutional credibility. The findings reinforce the relevance of signaling theory by showing that customer trust plays an important role in reducing uncertainty and strengthening users’ acceptance of AI-enabled banking services. In digital banking environments characterized by information asymmetry and algorithmic opacity, users appear to rely heavily on perceptions of security, transparency, and reliability when deciding whether to adopt AI-driven financial technologies. This suggests that trust functions not only as a direct predictor of behavioral intention, but also as an important mechanism shaping users’ attitudes toward AI-enabled banking services. The study also strengthens Media Equation Theory by demonstrating that anthropomorphic characteristics of AI positively influence both customer attitude and behavioral intention. Human-like interaction features such as conversational communication, empathy, and personalized responses may reduce psychological distance and increase users’ emotional comfort when interacting with AI-enabled banking services. Another important contribution is the insignificant moderating effect of social influence, which suggests that users in high-risk financial contexts rely more on personal evaluations and trust perceptions than on external social pressure when making AI adoption decisions. Overall, the findings suggest that AI adoption in high-risk financial environments should be understood as a psychologically and relationally driven process rather than solely as a utilitarian technology acceptance process.

### 6.2 Practical implications

This study provides several practical implications for financial institutions, fintech companies, AI developers, and policymakers seeking to increase customer adoption of AI-enabled digital banking services. The findings suggest that successful AI adoption depends not only on technological sophistication, but also on users’ perceptions of trustworthiness, emotional comfort, and institutional credibility. First, the significant role of customer trust indicates that financial institutions should prioritize trust-building strategies when implementing AI-enabled banking services. Banks and fintech companies should strengthen transparency regarding AI operations, data protection, cybersecurity practices, and ethical AI governance to reduce customer uncertainty and increase confidence in AI-driven financial technologies. Since trust significantly influences both customer attitude and behavioral intention, improving institutional credibility becomes essential for encouraging long-term AI adoption.

Second, the findings highlight the importance of anthropomorphic AI characteristics in shaping customer acceptance. Financial institutions should therefore design AI systems with more human-like interaction capabilities such as conversational interfaces, personalized communication, and responsive interaction styles. These features may help reduce psychological distance, improve emotional comfort, and create more engaging user experiences in digital banking interactions. Finally, because customer attitude was found to be the strongest predictor of behavioral intention, financial institutions should focus on providing secure, trustworthy, emotionally comfortable, and user-friendly AI service experiences. The findings also suggest that strategies emphasizing trust, security, transparency, and credibility may be more effective than relying mainly on social influence or peer endorsement in encouraging AI adoption within high-risk financial environments.

## 7 Limitations and future research

Despite providing important insights into the determinants of behavioral intention toward AI-enabled digital banking services, this study has several limitations that should be acknowledged. First, the study employed a cross-sectional research design, which limits the ability to examine changes in users’ perceptions and behavioral intentions over time. Since trust, customer attitude, and perceptions toward AI technologies may evolve as users gain more experience with AI-enabled banking services, future studies are encouraged to apply longitudinal approaches to better understand the dynamic nature of AI adoption behavior. Second, this study focused primarily on users of digital banking services in a specific national context. As perceptions of AI, trust, and technology adoption may vary across cultural, economic, and regulatory environments, the generalizability of the findings may be limited. Future research could conduct cross-country or cross-cultural comparisons to examine whether the relationships identified in this study remain consistent across different financial and technological environments. In addition, this study did not conduct subgroup or measurement invariance analysis, which may provide further insights into whether AI adoption mechanisms differ across demographic or contextual groups.

Third, although this study integrated customer trust, anthropomorphic characteristics of AI, customer attitude, and social influence into a single framework, other potentially important variables were not included in the model. Factors such as perceived risk, perceived usefulness, AI literacy, privacy concerns, and transparency of AI systems may also influence behavioral intention toward AI-enabled banking services. Future studies are therefore encouraged to expand the research model by incorporating additional psychological, technological, and contextual variables to provide a more comprehensive understanding of AI adoption in financial services.

Another limitation concerns the insignificant moderating effect of social influence. Although the findings suggest that users in high-risk financial environments rely more on personal evaluations and trust perceptions than on social pressure, the role of social influence may vary depending on demographic characteristics, technological familiarity, or specific types of AI-enabled financial services. Future research could therefore examine moderation effects across different user segments or conduct multi-group analysis to better understand contextual variations in AI adoption behavior.

Finally, this study relied on self-reported questionnaire data, which may be subject to response bias and individual perception limitations. Although common method bias and measurement validity were assessed, future studies may benefit from combining survey methods with experimental, observational, or behavioral usage data to strengthen the robustness of findings related to AI-enabled banking adoption.

## Data Availability

Publicly available datasets were analysed in this study. This data can be found here: https://zenodo.org/records/20685201.
